# Is there association between hyperdense middle cerebral artery sign on CT scan and time from stroke onset within the first 24-hours?

**DOI:** 10.1186/s12883-015-0358-5

**Published:** 2015-07-03

**Authors:** James Haridy, Leonid Churilov, Peter Mitchell, Richard Dowling, Bernard Yan

**Affiliations:** Royal Melbourne Hospital, Melbourne, Australia; Florey Institute of Neuroscience and Mental Health, Melbourne, Australia; Department of Radiology, Neurointervention Service, Royal Melbourne Hospital, Parkville, Victoria, 3050 Australia; Department of Medicine, Melbourne Brain Centre, Royal Melbourne Hospital, University of Melbourne, Melbourne, Australia

**Keywords:** Ischaemic Stroke, Hyperdense artery sign, Susceptibility vessel sign, Stroke imaging, Neuroimaging, Acute stroke

## Abstract

**Background:**

The hyperdense artery sign (HAS) on CT brain scan is an assumed radiological marker of acute intra-arterial thrombotic occlusion. However, the relationship between HAS between time of stroke onset has not been adequately investigated, leading to uncertainty regarding its validity as a marker of acute ischaemia. We attempted to determine if the presence of the hyperdense artery sign is associated with time from stroke onset.

**Methods:**

Retrospective cross-sectional study conducted in a tertiary referral centre. Consecutive patients with acute ischaemic stroke and confirmed middle cerebral arterial occlusion on initial CT angiogram from 2007–2011 were included. Visual estimation and manual measurement of Hounsfield units of affected and corresponding non-affected artery on non-contrast CT was completed and mean density was calculated from four separate readings. Primary outome measures were Time from stroke onset and HAS on both visual estimation and the ratio of mean value in Hounsfield Units (HU) of affected to non-affected artery.

**Results:**

One hundred and fifty-four subjects with confirmed arterial occlusion on CT Angiogram were included in the study. There were no significant differences in age distribution or vascular risk factor presence between subjects with or without HAS. Subjects with HAS were less likely to be male (50.9 % vs 70.8 %, *p* = 0.02).) HAS was found in 106 (68.8 %) of all subjects. Median NIHSS score at presentation was significantly higher in the HAS group (17 vs 12, *p* = 0.02). No statistically significant association between HAS and stroke onset time or density ratio between affected and non-affected artery was detected overall within either the first 24-h or on subgroup analysis of those in the first 4.5-h. A small subgroup of three patients with stroke onset greater than 24-h all had absent HAS.

**Conclusions:**

No evidence of a correlation between time of stroke onset and presence of a HAS within the first 24-h post acute ischaemic stroke was identified. The HAS was associated with a higher NIHSS score at presentation.

## Background

Despite the development of newer imaging modalities, non-contrast computed tomography (NCCT), given its wide accessibility, remains the principal imaging technique in suspected acute stroke. The hyperdense artery sign (HAS) was first described in 1983 as a radiological marker of intra-arterial thrombotic occlusion and, although not definitively unproven, was possibly an early sign of acute ischaemic stroke [7]. Although HAS is widely described in the middle cerebral artery, termed Hyperdense Middle Cerebral Artery Sign (HMCAS), it is also reported in internal carotid artery, posterior cerebral artery, basilar artery and to a lesser extent, anterior cerebral artery [2, 5, 6, 8–10, 15, 20].

**Fig. 1:**
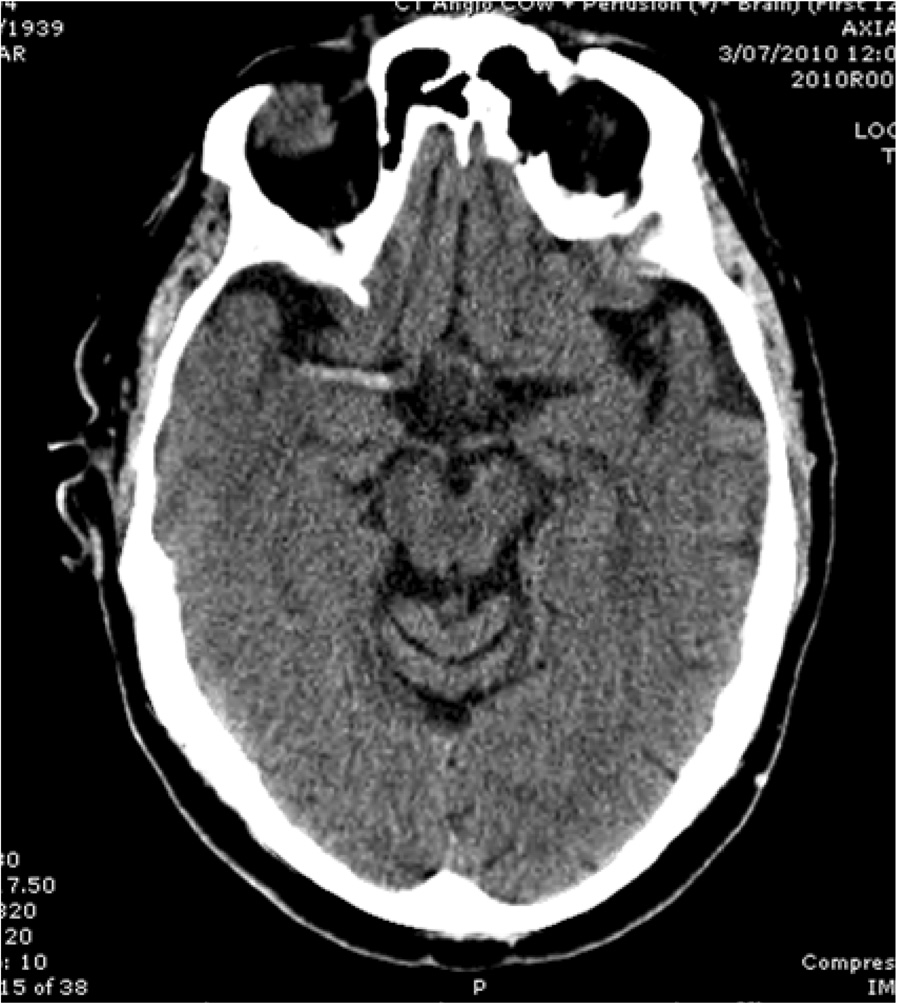
R MCA Hyperdense artery sign

HAS is defined as region of hyperdensity in comparison to the artery on the contralateral side, as seen on NCCT (Figure 1). This has been reported to be present on NCCT in 75 % of acute ischaemic stroke within the first 90 min of symptom onset [14, 24, 25]. The overall prevalence of HMCAS in acute ischaemic stroke varies in published studies between 5-75 % with the largest systematic review indicating a prevalence of around 25 % [28]. The vast majority of studies to date define a hyperdense artery as one that is denser than its contralateral counterpart when assessed by one or more neuroradiologists [3, 10, 15, 16, 20, 28].

Recent evidence suggests that clot density on NCCT reflects the composition of the clot [17]. Normally flowing blood column is characterized by a Hounsfield value of approximately 40 units, where an acute clot within a vascular structure averages closer to 61–80 units [17, 19]. As the acute thrombus progresses, its composition changes from RBC-rich ‘red thrombus’ to a predominately fibrin-based ‘white thrombus’. It has been postulated that the extrusion of serum in an acute thrombus, and subsequent increased haemoglobin concentration, is the cause of the increase in density on NCCT [19]. Kirchhof et al. showed that white thrombi had a CT attenuation of 24 +/− 8 HU compared to red thrombi at 76 +/− 9 HU, with a linear relationship between haematocrit level and CT attenuation [13]. Presumably it also reflects the acuity of the thrombus, although further research is required to determine the specific relationship between time from symptom onset and the presence of the HAS. A non-significant relationship has been noted on previous studies, although this has not been examined as a primary outcome [15, 20]. Despite this, prior studies involving intra-arterial thrombolysis have used the HAS and it’s subsequent disappearance as both a selection criteria and outcome measure to define successful recanalization in the first 24-h.

The potential benefits of further imaging guidance to identify onset time is pertinent in the time critical setting of potential thrombolysis within the first 4.5-h following stroke. Although advanced imaging systems examining penumbra and perfusion show promise they are still not available in all centres. In this study, we aimed to establish the relationship between stroke onset time and the HAS.

## Methods

### Participants

All patients presenting with an ischaemic stroke between 2007–2011 to the Royal Melbourne Hospital stroke care unit who had both NCCT (4.5 mm slices) and CT-Angiogram (CTA) on admission were retrospectively identified from the stroke database. Patients imaging was reviewed to identify those who had confirmed vessel occlusion on CTA that corresponded clinically with presenting symptoms. Those who either had haemorrhagic strokes or no confirmed arterial occlusion on CTA were excluded from the study as were those with strokes not affecting the Middle Cerebral Artery (MCA). Demographic and stroke risk factors including hypertension, diabetes, age, smoking history, history of atrial fibrillation, hypercholesterolaemia were extracted from the stroke database. Ethics approval was granted via the human research ethics committee prior to commencement.

### Procedures

All NCCTs were reviewed by a blinded assessor (JH) to identify the Hyperdense Middle Cerebral Artery (HMCA) defined as an MCA denser than its contralateral counterpart including the ‘dot’ sign of the MCA in the sylvian fissure. All hyperdense signs carried the additional criteria of disappearance of the increased density on bone window settings. Four random 1 mm^2^ spot measurements in Hounsfield Units (HU) were taken from the highest density area on visual estimation of the affected artery and the mean of these values used, with similar measurements taken from the corresponding non-affected artery on the contralateral side.

Following identification of the HAS, the corresponding CTA was then reviewed. Cases were excluded where a corresponding arterial occlusion was not identified on CTA at the site of the HAS. A randomized subset of two-third of all NCCTs and corresponding CTAs were reviewed independently by a second blinded neuroradiologist (one of BY, PJM or RJD). The interobserver agreement for the HAS and occlusion was assessed using Cohen’s kappa statistics.

Time of stroke onset data was collected and stored in the stroke registry. Where a wake-up stroke occurred, time of stroke onset was measured as midnight.

### Data analysis

Statistical analyses were performed using Stata IC v12 software. Data are presented as means and standard deviations or medians and interquartile range for continuous variables and as counts and percentages for categorical variables. Univariate comparisons of various factors of interest between HAS and non-HAS groups were made using Wicoxon-Mann–Whitney ranksum test or Fisher’s exact test depending on the nature of the underlying distribution. The differences in time from onset of stroke symptoms in patients with and without HAS were assessed using Wicoxon-Mann–Whitney ranksum test, while the magnitude of association between time from onset of stroke symptoms and the ratio of artery density between affected/non-affected arteries was assessed using Spearman correlation coefficient. For all statistical analyses, the significance level was set at a p < 0.05.

## Results

A total of 154 subjects with acute ischaemic stroke and corresponding MCA arterial occlusion on CTA were included in the in the study. Of these, 106 (68.8 %) had a HAS and 7 (4.5 %) were wake-up strokes. There were no significant differences in age distribution or vascular risk factor presence between subjects with or without HAS. There was a significantly higher proportion of males in the group with an absent HAS (70.8 % vs 50.9 %, *p* = 0.02). Median admission NIHSS scores in the HAS group (17 points, interquartile range 10 – 21) were significantly greater than in the non-HAS group (12 points, interquartile range 7–18, *p* = 0.02). Mean discharge Modified Rankin Score was significantly higher in the HAS group (3.9 vs 3.2, *p* = 0.01). The baseline and summary characteristics of the subjects are listed in Table 1.

**Table 1:** Summary statistics for patients with and without hyperdense artery sign

Characteristic	Patients with HAS	Patients without HAS	*P* Value
Number, n(%)	106 (68.8)	48 (31.2)	
Age, mean (SD), y	69.7	68.4	0.62
Male, n (%)	54 (50.9)	34 (70.8)	0.02
Vascular Risk Factors, n (%)			
Hypertension	62 (58.5)	29 (60.4)	0.82
Diabetes Mellitus	23 (21.7)	12 (25.0)	0.65
Cholesterol, mean	4.49 mmol/L	4.60 mmol/L	0.72
Atrial Fibrillation	40 (37.7)	16 (33.3)	0.6
Smoking	24 (22.6)	11 (22.9)	0.97
Ischaemic Heart Disease	21 (19.8)	16 (33.3)	0.07
Previous stroke	12 (11.3)	5 (10.4)	0.87
NIHSS Score admission, median (range)	17 (0–39)	12 (0–27)	0.02
Stroke onset to NCCT, median (range)	134 (52–1092)	126 (34–997)	0.76
Ratio affected:non-affected artery, median (range)	1.44 (1.09–2.00)	1.02 (0.84-1.47)	0.001
Mean artery density affected artery (HU)	59.0	42.9	
Death, n (%)	11 (10.4)	2 (4.2)	0.08
MRS at discharge, mean (range)	3.9 (0–6)	3.2 (0–6)	0.01

Interrater agreement on review of 102 scans (66 % of total) by a second neuroradiologist of a randomised subset of subjects for visual estimation of the HAS was moderate, with a kappa value 0.60.

Within the first 24-h, the median time from onset of stroke symptoms in patients with HAS was 134 min (interquartile range 89–263 min) and without HAS 126 min (interquartile range 85 – 345 min), resulting in the absence of the evidence of statistically significant association between the HAS on visual estimation and time from stroke within the first 24-h (*p* = 0.70). Ratio of artery density between affected/non-affected arteries similarly was not significantly associated with time from stroke onset within the first 24-h (Spearman’s Rho = −0.8; *p* = 0.42).

On subgroup analysis comparing 118 subjects presenting within the first 4.5-h post stroke onset with 36 subjects presenting 4.5-24 h post onset, there was no significant association between presence or absence of the HAS on visual estimation (Fisher’s exact *p* = 0.83). Ratio of artery density between affected/non-affected arteries was not significantly associated with presenting prior to 4.5-h vs 4.5-24 h (Wilcoxon-Mann–Whitney *p* = 0.42).

**Fig. 2:**
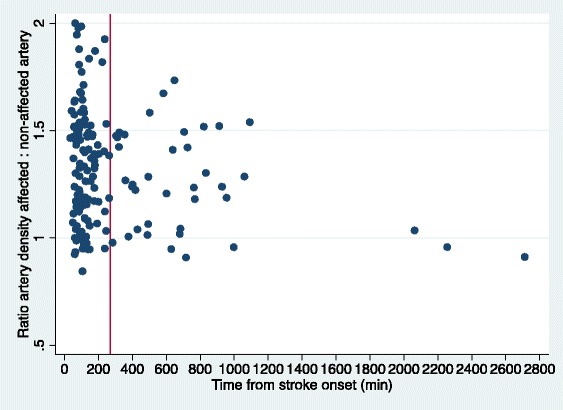
Ratio of affected to non-affect mean artery density by time from stroke onset with vertical line displaying 4.5-h mark

Figure 2 displays a scatterplot of the ratio to time from stroke onset. An additional three subjects were excluded due to presenting greater than 24-h post stroke onset. All of these subjects had an absent HAS. There were no significant differences in age distribution, sex ratio or presence of vascular risk factors between the groups less than/greater than 24-h post stroke onset.

## Discussion

Accurate determination of stroke onset time has gained increasing importance with the advent of time-dependent thrombolysis. The benefits on thrombolysis in the first 4.5 h has increased the necessity of objective means to assist in determining stroke onset, especially in patients who are unable to communicate or remember the onset of stroke symptoms (such as wake-up strokes). Newer imaging techniques such as diffusion-weighted MRI and CT-perfusion have yet to show clear guidance as to specific stroke onset time, and are available only in larger centres.

Our study further attempted to define the relationship between the HAS and stroke onset time. Previous studies have consistently found the HAS to be an early sign of ischaemia [6, 8, 10, 15, 20]. The association between time of stroke onset, imaging time and presence of HAS, however has not been studied as a primary outcome.

The HMCAS has consistently shown to be associated with earlier presentation [22], poor clinical outcome and prognosis [11, 18, 24, 27], severe neurological deficits and larger stroke territory [18, 24, 27]. Initial NIHSS/MRS scores and outcome at three-months are significantly worse in patients presenting with HMCAS [8, 11, 16, 18, 24, 27, 28]. Our study similarly displayed a significantly higher NIHSS score at admission with a present HAS as well as poorer clinical outcome as evidenced by higher discharge Modified Rankin Score. There was a trend towards greater mortality with a HAS in our study however this was not statistically significant.

Although clot density on NCCT has been reported to decrease in a linear fashion in comparison to time, this has not been specifically studied in relation to time from symptom onset as a primary outcome [4]. In a large study of the SITS register by Kharitonova et al., representing 1905 subjects with a HMCAS at presentation, 48 % had a disappearing HMCAS at 22–36 h after thrombolysis [12]. Those with a disappearing HMCAS on follow-up scan were significantly younger, less likely to have initial CT signs and had milder strokes as measured by initial NIHSS score. Early improvement in NIHSS was a significant predictor for disappearing HMCAS [12]. It is uncertain from the literature as to whether a disappearing HMCAS represents recanalization vs change in clot composition and likewise a persisting HMCAS may not always equate with continued occlusion [12, 22, 26]. Our finding of a higher NIHSS with the HAS, consistent with other studies, may be associated with larger or more prolonged arterial occlusion. The natural history of the HMCAS and rate of disappearance has not been specifically examined to date.

We found that a present HAS (by visual estimation) may be associated with time only on a small subset of patients 24-h post stroke onset. Our findings also show that the HAS is not associated with earlier presentation, although notably further studies examining subjects presenting greater than 24-h would be beneficial. Previous studies have reported a prevalence of hyperdense MCA sign at 75 % in the first 3-h and in 15 % from hours 12–24. Studies with hyperdense PCA and basilar signs have yielded similar results [15].

Similarly, there was no association between ratio of affected: non-affected mean artery density and time from stroke onset within the first 24-h. Prior studies have reported significantly reduced false positive rates when using measurement of absolute attenuation of affected and normal vessels, using a ratio of > 1.2 [14]. Our finding of a significantly reduced ratio in the subgroup of patients greater 24-h post stroke, although modest, may assist as a guide to determining stroke onset.

We also analysed a subgroup of patients presenting within the first 4.5-h post stroke onset, given accurate determination of stroke onset time in this period is clinically applicable to eligibility for thrombolysis. This subgroup did not display a statistically significant association with either presence of the HAS or arterial density ratio in comparison to those presenting 4.5-24 h post stroke onset.

More recently the HAS has been used as an element of functional outcome predictor scores (the DRAGON criteria) and a prognostic marker for thrombolysis [1, 23]. The HAS is a common inclusion in the development of functional outcome predictors scores for patients who receive thrombolysis [1, 23]. The lack of time-dependency of the HAS that was shown in our study should be considered in the use of these measures.

There were a number of limitations to our study. The vast majority of scans in our study were taken on 4.5 mm thick slice NCCT, with thin-slice NCCT only recently initiated on a regular basis in our institution. Recent evidence suggests that thin-slice NCCT allows more reliable and sensitive detection of arterial occlusion and has significantly higher inter-rater reliability [21]. Further studies using thin-slice NCCT may yield further information regarding the usefulness of HAS in predicting time from stroke onset.

## Conclusion

We showed that the likelihood of HAS in acute ischaemic stroke is not associated with time from onset of stroke within the first 4.5 or 24-h. Ratio of absolute artery density between affected and non-affected artery similarly is not associated with time from stroke onset. The HAS was associated with a higher NIHSS score at presentation.

## References

[1] Arnold M, Nedeltchev K, Schroth G, Baumgartner RW, Remonda L, Loher TJ (2004). Clinical and radiological predictors of recanalization and outcome of 40 patients with acute basilar artery occlusion treated with intra-arterial thrombolysis. J Neurol Neurosurg Psychiatry.

[2] Bamford J, Sandercock P, Dennis M, Burn J, Warlow C (1991). Classification and natu- ral history of clinically identifiable subtypes of cerebral infarction. Lancet.

[3] Bastianello S, Pierallini A, Colonnese C, Brughitta G, Angeloni U, Antonelli M (1991). Hyperdense middle cerebral artery CT sign. Comparison with angiography in the acute phase of ischemic supratentorial infarction. Neuroradiology.

[4] Berge E, Nakstad PH, Sandset PM (2001). Large middle cerebral artery infarctions and the hyperdense middle cerebral artery sign in patients with atrial fibrillation. Acta Radiol.

[5] Connell L, Koerte IK, Laubender RP, Morhard D, Linn J, Becker HC (2012). Hyperdense basilar artery sign-a reliable sign of basilar artery occlusion. Neuroradiology.

[6] Ehsan T, Hayat G, Malkoff MD, Selhorst JB, Martin D, Manepalli A (1994). Hyperdense basilar artery. An early computed tomography sign of thrombosis. J Neuroimaging.

[7] Gacs G, Fox AJ, Barnett HJ, Vinuela F (1983). CT visualization of intracranial arterial thromboembolism. Stroke.

[8] Goldmakher GV, Camargo EC, Furie KL, Singhal AB, Roccatagliata L, Halpern EF (2009). Hyperdense basilar artery sign on unenhanced CT predicts thrombus and outcome in acute posterior circulation stroke. Stroke.

[9] Herrera M, Erro ME, Gallego J (2007). Hyperdense posterior cerebral artery sign. Neurologia.

[10] Jensen UR, Weiss M, Zimmermann P, Jansen O, Riedel C (2010). The hyperdense anterior cerebral artery sign (HACAS) as a computed tomography marker for acute ischemia in the anterior cerebral artery territory. Cerebrovasc Dis.

[11] Kharitonova T, Ahmed N, Thoren M, Wardlaw JM, von Kummer R, Glahn J (2009). Hyperdense middle cerebral artery sign on admission CT scan--prognostic significance for ischaemic stroke patients treated with intravenous thrombolysis in the safe implementation of thrombolysis in Stroke International Stroke Thrombolysis Register. Cerebrovasc Dis.

[12] Kharitonova T, Thoren M, Ahmed N, Wardlaw JM, von Kummer R, Thomassen L (2009). Disappearing hyperdense middle cerebral artery sign in ischaemic stroke patients treated with intravenous thrombolysis: clinical course and prognostic significance. J Neurol Neurosurg Psychiatry.

[13] Kirchhof K, Welzel T, Mecke C, Zoubaa S, Sartor K (2003). Differentiation of white, mixed, and red thrombi: Value of CT in estimation of the prognosis of thrombolysis-phantom study. Radiology.

[14] Koo CK, Teasdale E, Muir KW (2000). What constitutes a true hyperdense middle cerebral artery sign?. Cerebrovasc Dis.

[15] Krings T, Noelchen D, Mull M, Willmes K, Meister IG, Reinacher P (2006). The hyperdense posterior cerebral artery sign: a computed tomography marker of acute ischemia in the posterior cerebral artery territory. Stroke.

[16] Leys D, Pruvo JP, Godefroy O, Rondepierre P, Leclerc X (1992). Prevalence and significance of hyperdense middle cerebral artery in acute stroke. Stroke.

[17] Liebeskind DS, Sanossian N, Yong WH, Starkman S, Tsang MP, Moya AL (2011). CT and MRI early vessel signs reflect clot composition in acute stroke. Stroke.

[18] Manelfe C, Larrue V, von Kummer R, Bozzao L, Ringleb P, Bastianello S (1999). Association of hyperdense middle cerebral artery sign with clinical outcome in patients treated with tissue plasminogen activator. Stroke.

[19] New PFJ, Aronow S (1976). Attenuation measurements of whole blood and fractions in computed tomography. Radiology.

[20] Ozdemir O, Leung A, Bussiere M, Hachinski V, Pelz D (2008). Hyperdense internal carotid artery sign: a CT sign of acute ischemia. Stroke.

[21] Riedel CH, Zoubie J, Ulmer S, Gierthmuehlen J, Jansen O (2012). Thin-slic reconstructions of nonenhanced CT images allow for detection of thrombus in acute stroke. Stroke.

[22] Sharma VK, Venketasubramanian N, Teoh HL, Chan BP (2011). Hyperdense middle cerebral artery sign and stroke outcomes after intravenous thrombolysis. Cerebrovasc Dis.

[23] Strbian D, Meretoja A, Alhelm FJ, Pitkaniemi J, Lyrer P, Kaste M (2012). Predicting the outcome of IV thrombolysis- treated ischemic stroke patients: The DRAGON score. Neurology.

[24] Tomsick T, Brott T, Barsan W, Broderick J, Haley EC, Spilker J, Khoury J (1996). Prognostic value of the hyperdense middle cerebral artery sign and stroke scale score before ultraearly thrombolytic therapy. AJNR Am J Neuroradiol.

[25] Urbach H, Brechtelsbauer D, Klotz S, Bendszus M, Solymosi L (1997). Detect- ability of new medial infarcts by CT: appearance of ischemic signs. Rofo Fortschr Geb Rontgenstr Neuen Bildgeb Verfahr.

[26] van Overbeek EC, Knottnerus IL, van Oostenbrugge RJ (2010). Disappearing hyperdense middle cerebral artery sign is associated with striatocapsular infarcts on follow-up CT in ischemic stroke patients treated with intravenous thrombolysis. Cerebrovasc Dis.

[27] von Kummer R, Meyding-Lamade U, Forsting M, Rosin L, Rieke K, Hacke W, Sartor K (1994). Sensitivity and prognostic value of early CT in occlusion of the middle cerebral artery trunk. AJNR Am J Neuroradiol.

[28] Wardlaw JM, Mielke O (2005). Early signs of brain infarction at CT: observer reliability and outcome after thrombolytic treatment--systematic review. Radiology.

